# Modeling the Effect of Composition on Formation of Aerosolized Nanoemulsion System Encapsulating Docetaxel and Curcumin Using D-Optimal Mixture Experimental Design

**DOI:** 10.3390/ijms21124357

**Published:** 2020-06-19

**Authors:** Azren Aida Asmawi, Norazlinaliza Salim, Emilia Abdulmalek, Mohd Basyaruddin Abdul Rahman

**Affiliations:** 1 Integrated Chemical BioPhysics Research, Department of Chemistry, Faculty of Science, Universiti Putra Malaysia, Serdang 43400, Selangor, Malaysia; gs45976@student.upm.edu.my (A.A.A.); azlinalizas@upm.edu.my (N.S.); emilia@upm.edu.my (E.A.); 2 UPM-MAKNA Cancer Laboratory, Institute of Bioscience, Universiti Putra Malaysia, Serdang 43400, Selangor, Malaysia

**Keywords:** D-optimal, mixture experimental design, aerosol, curcumin, docetaxel, nanoemulsion, pulmonary delivery

## Abstract

The synergistic anticancer effect of docetaxel (DTX) and curcumin (CCM) has emerged as an attractive therapeutic candidate for lung cancer treatment. However, the lack of optimal bioavailability because of high toxicity, low stability, and poor solubility has limited their clinical success. Given this, an aerosolized nanoemulsion system for pulmonary delivery is recommended to mitigate these drawbacks. In this study, DTX- and CCM-loaded nanoemulsions were optimized using the D-optimal mixture experimental design (MED). The effect of nanoemulsion compositions towards two response variables, namely, particle size and aerosol size, was studied. The optimized formulations for both DTX- and CCM-loaded nanoemulsions were determined, and their physicochemical and aerodynamic properties were evaluated as well. The MED models achieved the optimum formulation for DTX- and CCM-loaded nanoemulsions containing a 6.0 wt% mixture of palm kernel oil ester (PKOE) and safflower seed oils (1:1), 2.5 wt% of lecithin, 2.0 wt% mixture of Tween 85 and Span 85 (9:1), and 2.5 wt% of glycerol in the aqueous phase. The actual values of the optimized formulations were in line with the predicted values obtained from the MED, and they exhibited desirable attributes of physicochemical and aerodynamic properties for inhalation therapy. Thus, the optimized formulations have potential use as a drug delivery system for a pulmonary application.

## 1. Introduction

Cancer is one of the leading causes of death globally, and lung cancer tops the mortality rate with the lowest survival rate among all the cancers [1]. Chemotherapy remains a major option for the treatment of lung cancer. Among the most potent of chemotherapeutic drugs used for lung cancer treatment are taxanes, which comprise docetaxel (DTX). Nevertheless, this type of treatment is always riddled with dose-limiting side effects and drug resistance issues [2,3]. Recent progress in natural products has paved the way for the discovery of a novel chemotherapy adjunct for cancer treatment. It has been seen that a compound with medicinal properties known as curcumin (CCM) can synergistically improve the anticancer activity of DTX [4,5,6]. Hence, the synergistic anticancer effect exhibited by DTX and CCM may potentiate the treatment of lung cancer and make them attractive therapeutic drug candidates. However, due to their hydrophobic nature, both DTX and CCM are usually associated with slow drug absorption and low stability leading to inadequate maximum bioavailability for the therapeutic response [7,8].

At this juncture, the parenteral administration of chemotherapeutics is a common route for treating lung cancer. The mechanism of chemotherapeutic drugs is based on systemic circulation, which requires the drugs to pass through several natural defense mechanisms before reaching the lungs. The non-specific distribution of the drugs across the human body may result in rapid clearance and poor pharmacokinetics [9]. Consequently, the focus of the present research was on an alternative route via the pulmonary, to mitigate the disadvantages of the current course. Direct deposition of drugs at the targeted region of the lungs can be achieved via inhalation therapy, which may be far more effective with increased local drug concentration. These contribute to the high systemic bioavailability, evasion of first-pass metabolism, and quick onset of drug action. Adverse effects caused by excessive dosing of drugs can also be avoided. Several types of respiratory diseases such as chronic obstructive pulmonary diseases (COPD), bronchitis, and asthma also have been conventionally treated via this route [10].

Nevertheless, due to the complexity of lung structure and its clearance mechanisms, inhalation of drug solution may not be efficiently delivered to the deep lung region, where the tumors are typically found. Besides, the lack of optimal DTX and CCM bioavailability due to low aqueous solubility and stability has hampered their progress towards better development and clinical use. The appropriate drug carrier system to encapsulate the drugs without affecting the drugs primary bioactive is, therefore, highly required for exploiting the nature of respiratory transportation. The availability of safe and biocompatible lipids as per safety standards along with the potential to enhance drug bioavailability makes nanoemulsion an ideal carrier for these hydrophobic drugs. The focal interest in formulating this aerosolized nanoemulsion system is on the presence of lipophilic core in the nanosized system, which can improve the solubility, stability, dissolution rate, and uniform distribution of drugs in the alveoli region because of the increased surface contact area [11,12].

A large number of compositions used in a formulation may result in irritancy problems and dose-related toxicity when applied for pulmonary delivery. Somehow, screening of random formulations is inadequate to produce nanoemulsion at the minimal optimal concentration with the desired properties, and hence, optimizing the nanoemulsion system is equally important. D-optimal mixture experimental design (MED) utilizes a modeling technique that considers the interaction effects. It consists of a collection of mathematical and statistical methods that match the experimental data with polynomial equations [13,14], thereby explaining the dataset’s behavior. Additionally, the evaluation of the model’s interaction involves a minimum number of experimental runs, which can reduce the time consumption and expense. This work aims to determine the optimized formulation of aerosolized DTX- and CCM-loaded nanoemulsions based on the best-fitted model suggested by MED. The optimization models presented in this study allow us to understand how the independent and response variables interact to form the nanoemulsion system and offer insight into the degree to which model terms have significantly influenced the formation process.

## 2. Results and Discussion

### 2.1. Fitting the Models

The effects of five independent variables, namely palm kernel oil ester (PKOE): safflower seed oil (A), lecithin (B), Tween 85:Span 85 (C), glycerol (D), and water (E), on particle size and volume median diameter (VMD) were explored using the D-optimal mixture experimental design (MED) for both DTX- and CCM-loaded nanoemulsions. The upper and lower limits of the independent variables were obtained from previous data collected during the preliminary study [15], while the software automatically calculated the amount of water. The candidate points referring to the design space and response values for both DTX- and CCM-loaded nanoemulsions are depicted in Table 1. The obtained data were fitted into three models, namely linear, quadratic, and special cubic models. Among them, the quadratic polynomial model was found to be the best-fitted to the experimental data of particle size. In contrast, the linear polynomial model matched with the experimental data of volume median diameter (VMD) for both DTX- and CCM-loaded nanoemulsion formulations.

**Figure 1:**
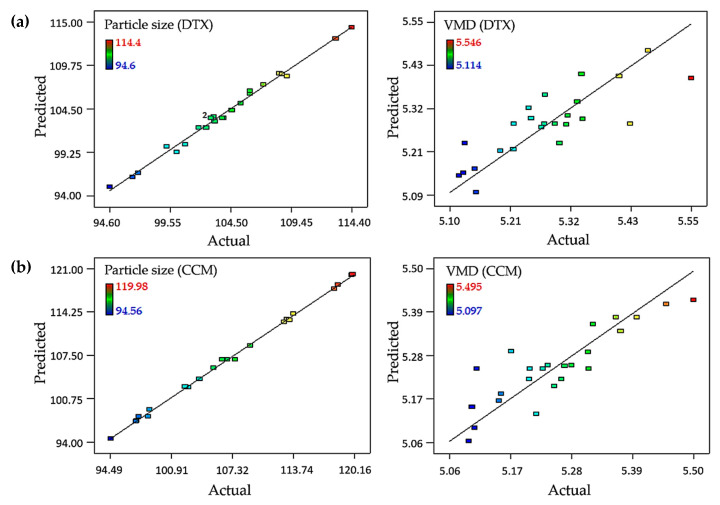
Scatter plot of predicted versus actual for (**a**) DTX-loaded nanoemulsion and (**b**) CCM-loaded nanoemulsion towards particle size and aerosol size (VMD).

The correlation coefficient (*R*^2^) values for the particle size of the DTX- and CCM-loaded nanoemulsions were 0.9922 and 0.9984, respectively, while the *R*^2^ values for VMD were 0.6894 and 0.7610, respectively. These values specified that the model could be predicted up to 99.22 and 99.84%, respectively, of the particle size observed value, leading to a strong correlation between the predicted data and actual values. The VMD observed value could only be predicted up to 68.94 and 76.10% for the DTX- and CCM-loaded nanoemulsions, respectively. These findings are illustrated by the linearity plot between the predicted and actual values of particle size and the VMD. Good-fitting regression models for particle size were obtained compared to the VMD for both formulations (Figure 1). The adequate precision for the particle size of the DTX- and CCM-loaded nanoemulsions were 35.58 and 64.87, respectively, whereas the adequate precision for VMD was 11.80 and 13.36, respectively. The signal-to-noise ratio of the models was appropriate because of the high value of adequate precision (>4.00), which indicated adequate model discrimination, as it compared the range of the predicted values at the design points to the average prediction error [16,17].

The predicted values for each response in both formulations were derived using regression Equations (1) and (2) for DTX-loaded nanoemulsion and regression Equations (3) and (4) for CCM-loaded nanoemulsion, as suggested by ANOVA. The generated mathematical models were proven to be significant and valid for the responses, as they had small *p*-values (*p* < 0.0001) and large *F*-values, which stipulated a good match between the regression models and experimental data. The models are significant if the *p*-value is below 0.05, suggesting that the model is significant at the probability level of 95% [13,18]. Other than that, a lack of fitness of the models was not significantly related to pure error (*p* > 0.05). Following *p*-values of <0.05, the interactions of AB, AC, AD, AE, CD, and CE harbored significant effects on the particle size of a DTX-loaded nanoemulsion, while interactions of AB, AC, AD, AE, BC, BD, BE, CE, and DE showed a significant effect on the particle size of a CCM-loaded nanoemulsion. The ANOVA results for both formulations are summarized in Table 2.

DTX-loaded nanoemulsion:

(1) $$\begin{array}{cc} \mathrm{Particle}\;\mathrm{size} & =\;128.29A+208.83B-219.77C+133.96D+114.30E-191.85\mathrm{AB}\\ & +\;350.39\mathrm{AC}-96.55\mathrm{AD}-54.91\mathrm{AE}+115.41\mathrm{BC}-79.11\mathrm{BD}-126.94\mathrm{BE}\\ & +\;349.42\mathrm{CD}+336.19\mathrm{CE}-49.55\mathrm{DE} \end{array}$$

(2) Aerosol size (VMD)=5.55A+5.99B+4.60C+5.17D+5.21E

CCM-loaded nanoemulsion:

(3) $$\begin{array}{cc} \mathrm{Particle}\;\mathrm{size} & =\;168.39A+244.63B+149.55C+28.09D+118.53E-184.49\mathrm{AB}\\ & -\;327.76\mathrm{AC}+90.15\mathrm{AD}-99.98\mathrm{AE}-438.80\mathrm{BC}-168.24\mathrm{BD}\\ & -\;129.62\mathrm{BE}+29.91\mathrm{CD}-102.07\mathrm{CE}+123.59\mathrm{DE} \end{array}$$

(4) Aerosol size (VMD)=5.48A+6.06B+5.29C+4.91D+5.10E

### 2.2. Influence of Independent Variables on Particle Size

**Figure 2:**
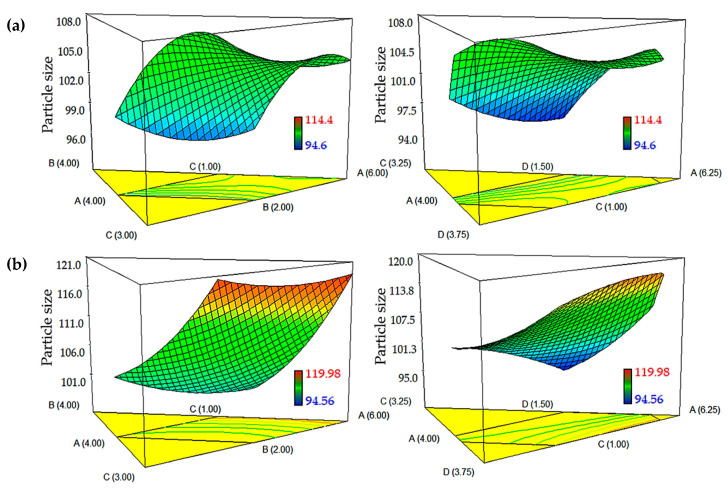
Graphical plots of (**a**) DTX-loaded nanoemulsion and (**b**) CCM-loaded nanoemulsion compositions toward particle size. The models show the interaction between independent variables including A: PKOE and Safflower seed oil mixture (1:1; *w*/*w*), B: lecithin, C: surfactant mixture of Tween 85 and Span 85 (9:1; *w*/*w*), and D: glycerol, while water was kept constant.

The DTX- and CCM-loaded nanoemulsions showed an increase in the particle size when there was an increase in the amount of oil (Figure 2). This effect was attributed to the increase of the dispersed phase’s viscosity in conjunction with the increase of oil content, which subsequently resulted in the increase of flow resistance and restriction on the droplet breakup rate [19]. This effect could be correlated with increased frequency of collision between the nanoemulsion particles, accompanied by an increase in the frequency of coalescence, resulting in a higher chance of coalescence between the particles [20]. Moreover, adding emulsifiers, including lecithin and Tween 85:Span 85 in the two formulations showed the opposite effect, whereby the particle size decreased when the surfactant and co-surfactant were increased. Other researchers have observed similar results, stating that the decrease of particle size could be due to the availability of free emulsifier to spontaneously enclose the surface of any new oil droplet formed during homogenization [18,20,21]. Moreover, the interfacial tension between water and oil may get decreased, which can reduce the stress for droplet breakup.

The addition of glycerol in the nanoemulsion system could decrease the particle size, as it contained both hydrophilic and lipophilic components in the structure that could invade the surfactant monolayer, resulting in a change in the system’s packing geometry, interfacial tension and surfactant flexibility [22,23]. However, if compared with the CCM-loaded nanoemulsion plot, the particle size for the DTX-loaded nanoemulsion was slightly increased initially, as the concentration of emulsifiers and glycerol increased, and the concentration of oil decreased. It started to decline when the concentration of emulsifiers was set to increase, whereby the DTX-loaded nanoemulsion would reach a consistent level of CCM-loaded nanoemulsion in the graphical plot. This finding might be attributed to the higher lipophilicity of DTX (logP_O/W_ = 4.1) compared to CCM (logP_O/W_ ~ 3.0), which observed a reduction of emulsification efficiency at low concentration of emulsifiers and high concentration of oil.

### 2.3. Influence of Independent Variables on Aerosol Size

**Figure 3:**
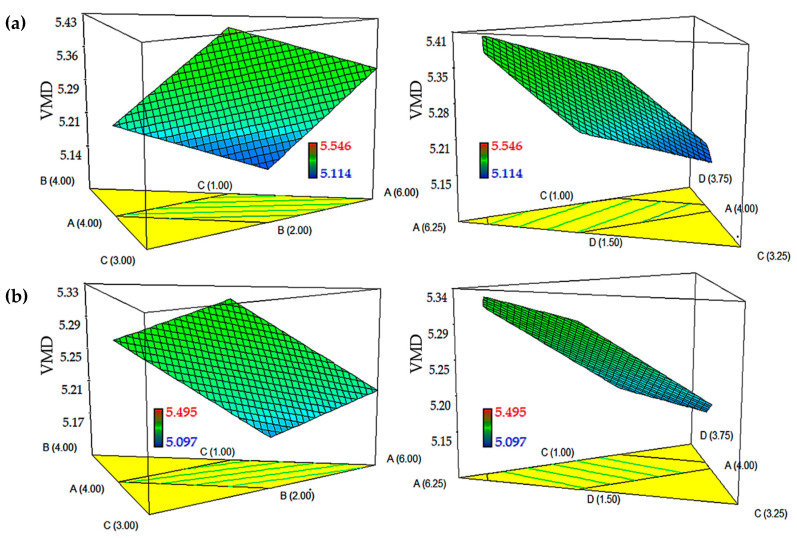
Graphical plots of (**a**) DTX-loaded nanoemulsion and (**b**) CCM-loaded nanoemulsion compositions toward VMD. The models show the interaction between independent variables including A: PKOE and Safflower seed oil mixture (1:1; *w*/*w*), B: lecithin, C: surfactant mixture of Tween 85 and Span 85 (9:1; *w*/*w*), and D: glycerol, while water was kept constant.

The aerodynamic diameter of nanoemulsion aerosols is the determinant of the deposition site in the lungs, as large particles (>6 μm) tend to deposit mainly in the upper airways. The production of aerosol with an aerodynamic diameter between 2 and 6 μm is found to be best-suited to treat central and small airways [24]. In this work, both models for DTX- and-CCM loaded nanoemulsions showed VMD values in the range of 5.0–5.6 μm. As illustrated in Figure 3, the VMD value decreased as the amount of Tween 80:Span 80 (9:1, *w*/*w*) and glycerol increased in both the DTX- and CCM-loaded nanoemulsions. This effect was obtained due to incorporation of surfactant and co-solvent in the system, which reduced the interfacial tension at the interface of oil and water by improving optimal curvature and lowering elasticity of the surfactant monolayer. Consequently, it decreased the viscosity of the system, as well [22]. However, the VMD value increased as the amount of glycerol increased and the amount of Tween 80:Span 80 (9:1, *w*/*w*) decreased because of the difficulty in breaking up the oil-water interface without decreasing interfacial tension [25].

Characteristics of the nanoemulsion system like viscosity and the overall surface tension would notably affect the system’s aerosolization efficiency. Increasing the viscosity or surface tension would need higher energy to atomize the nanoemulsion into aerosols, leading to an increase in aerosol size [26,27]. Arbain et al. and Shah et al. report nearly the same observations wherein decreasing the surface tension and viscosity of the nebulized nanoemulsion resulted in effective aerosolization using the Omron nebulizer [11,28]. Remarkably, the VMD value increased as the amount of oil and lecithin increased in DTX-loaded nanoemulsion. In contrast, the VMD value decreased as the amount of oil increased, and the amount of lecithin increased in the CCM-loaded nanoemulsion. This phenomenon might be due to lower lipophilicity and molecular weight of CCM compared to DTX, which permits a minimum packing geometry and requires less surfactant (lecithin) to reduce the surface tension. Besides, a higher molecular weight of lipophilic drugs has been reported to increase the viscosity of the solution and generate a larger-size of aerosols [29].

As shown in Table 3, the experimental response values for randomized and optimized formulations, as suggested by the MED, were juxtaposed with the predicted values to verify the generated models. The results showed that predicted values of all formulations in both the DTX- and CCM-loaded nanoemulsions resembled the experimental values closely. Moreover, the percentage of residual standard error (RSE) values were small with less than 2.2% for particle size and less than 3.8% for the VMD. These results were correlated reasonably well with the models’ regression correlation between the predicted and actual values, as higher *R*^2^ values were obtained for particle size compared to that of the VMD. The RSE values for both responses fell within the acceptable limits of model validation. It could be concluded that the postulated models were acceptable for use in predicting the response accurately.

### 2.4. Physicochemical and Aerodynamic Properties of Optimized Nanoemulsions

It is widely understood that nanoemulsion systems with a high quantity of oil, surfactants, and co-solvent may increase the safety issues in pulmonary application [30]. Therefore, it is necessary to use a minimal and optimal concentration of these components in the nanoemulsion formulation to allow the formation of a stable, safe, and fine nanoemulsion. In this study, the optimal formulation for a DTX-loaded nanoemulsion (DNE_Opt_) and CCM-loaded nanoemulsion (CNE_Opt_) were selected based on their minimum value of response variables at a minimal amount of nanoemulsion compositions for maximizing the effectiveness on the delivery and treatment of inhalable drugs. The optimized formulations for both DNE_Opt_ and CNE_Opt_ could be attained with a 6.0 wt% mixture of PKOE and safflower seed oils (1:1), 2.5 wt% of lecithin, 2.0 wt% mixture of Tween 85 and Span 85 (9:1), and 2.5 wt% of glycerol in the aqueous phase as suggested by MED.

**Figure 4:**
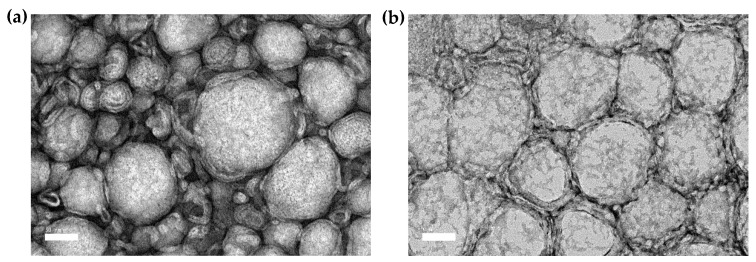
TEM images of (**a**) optimized DTX-loaded nanoemulsion (DNE_Opt_) and (**b**) optimized CCM-loaded nanoemulsion (CNE_Opt_) stained with uranyl acetate at 28 ± 1 °C. Images were observed under 50,000× magnification with scale bar = 50 nm.

Table 4 summarizes the overview of the physicochemical properties of the optimized formulations. The mean particle size of DNE_Opt_ and CNE_Opt_ was found to be 95.80 and 96.94 nm, respectively, with an RSE of less than 4%. The obtained particle size of the optimized formulations was further ascertained by the TEM micrographs, as depicted in Figure 4. The particles of the nanoemulsions were spherical, and the average particle size measured using TEM was in line with the average size determined from the photon correlation spectroscopy. The stability of the nanoemulsion system would increase when the polydispersity index (PDI) value was minimized, whereas the Ostwald ripening due to repulsive interaction between the particles during storage could be reduced when the *ζ*-potential value was maximized [31]. The PDI and *ζ*-potential values for both optimized formulations were in the range 0.22–0.25 and greater than −30 mV, respectively. These results suggest that the systems are slightly polydisperse but still adequate for pulmonary administration and they possess a sufficiently high negative surface charge to maintain stability due to the presence of anionic groups of fatty acids and glycol in the oils and lecithin [32,33].

Administration of too acidic or basic and hypo- or hyperosmotic formulations via the pulmonary route is known to cause cough and irritation of the lung linings, which can increase drug loss and reduce the carrier efficiency [34,35]. The formulation’s pH for liquid preparation intended for pulmonary administration should resemble closely with the pH of lung fluid at around 7.0 and 8.0 [36], while for human red blood cells, the normal osmotic pressure in the range of 280–320 mOsm/kg was measured at pH 7.40 [37]. The pH and osmolality values of the optimized formulations fell within the acceptable range for pulmonary delivery. On top of that, both formulations exhibited low viscosity values between 2.05 and 2.22 cP, which serve as a key parameter in influencing the effective delivery of drugs via the pulmonary route. The aerosolization efficiency of the formulations will be reduced at high viscosity as it contributes to an increase in aerodynamic diameter [26,27].

The laser diffraction method was used during the stage of aerosolization to assess the quality of the aerosol, while the Anderson cascade impaction (ACI) method was applied for summative aerosolization for inhalation. As given in Table 5, the optimized formulations had produced aerosol size within the respirable range, with the VMD (DV_50_) and mass median aerodynamic diameter (MMAD) falling within the optimal respirable range (2–6 µm). However, the size of the aerosol from the analysis of laser diffraction was relatively greater than that of the cascade impaction method, which was similar to the results obtained from other findings [28,38]. This effect was attributed to the loss of small particles during aerosolization through evaporation during the laser diffraction analysis, which led to a significant shift in the particle size distribution, causing a larger aerosol size than in the ACI method. Both DNE_Opt_ and CNE_Opt_ exhibited relatively narrow size distribution (<2.00) with span values of 1.64 and 1.69 and geometric standard deviation (GSD) values of 1.71 and 1.67, respectively.

**Figure 5:**
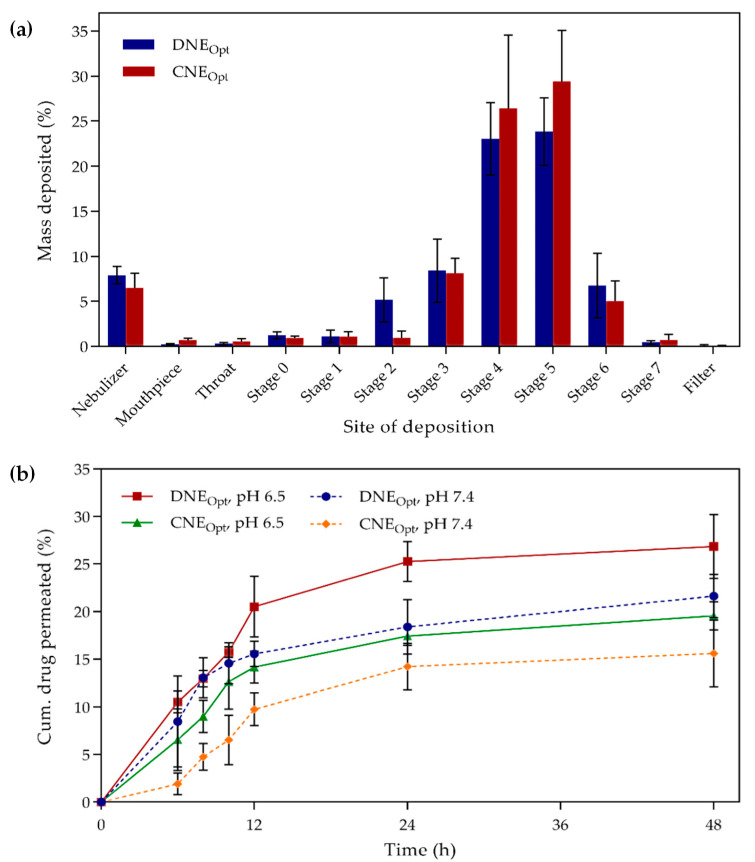
In vitro studies of (**a**) drug deposition using eight-stage Anderson cascade impactor and (**b**) drug release using the dialysis membrane diffusion method in simulated lung fluid (SLF) solution of pH 6.5 and 7.4 at a constant temperature of 37 ± 1 °C. Error bars denote standard deviation (*n* = 3).

For both the optimized formulations, the values of percent inhaled (PI) and percent dispersed (PD) were determined to be high since drug inhalation and emission into the lungs were more than 70%, while the rest of the drug may settle on the sampling apparatus. Similarly, a high percentage of fine particle fraction (FPF > 75%) was obtained, which indicated that the majority of the aerosol particles could be predominated in the deep lung region. The results appeared to conform with the in vitro deposition of optimized formulations on the ACI (Figure 5a). It was found that most of the nebulized aerosols were deposited on stages 3, 4, 5, and 6, which represented lower parts of respiratory airways with the aerodynamic cut-off diameters of 3.3–4.70, 2.10–3.3, 1.10–2.10, and 0.65–1.10 µm, respectively. Additionally, the in vitro drug release profile (Figure 5b) showed that the optimized formulations were able to release the DTX and CCM up to 48 h steadily. This was due to the additional time needed for the drug to permeate from the core of the oil droplet through the interfacial layer of surfactants before it could reach into the dissolution medium. The sustained release of encapsulated DTX and CCM can offer prolonged exposure at the target site and further enhance the efficacy of the pulmonary application.

Moreover, the drug release profiles of the encapsulated drugs were found to be relatively faster compared to the free drug solubilized in an aqueous solution, as reported by several studies [39,40,41], due to their poor water solubility. The drug release was lower in simulated lung fluid (SLF) at pH 7.4 compared to pH 6.5. These results were in agreement with previous studies on DTX and CCM, where the release was accelerated significantly under an intracellular tumor environment (pH~6.5) than an intracellular healthy environment (pH 7.4) [42,43]. The DNE_Opt_ was able to release the drug faster compared to CNE_Opt_ due to a lower degree of ionization in the CCM compared to DTX when tested at pH 6.5 and 7.4. According to the passive diffusion barrier concept, the drug in nonionized form could passively be diffused through the barriers effectively [44]. In contrast, the drug at a high degree of ionization state was deemed to be difficult in passing through the membranes, as it carried charge [45]. The result of optimized nanoemulsions with appropriate physicochemical and aerodynamic properties for inhalation and a sustained drug release manner might suggest the nanoemulsions great potential in pulmonary application.

## 3. Materials and Methods

### 3.1. Materials

Docetaxel (DTX, 98% purity) and trifluoroacetic acid (TFA, 99% extra purity) were purchased from Acros Organics (Fair Lawn, NJ, USA), while curcumin (CCM, 90% purity) and methanol (99.9% purity) were purchased from Merck (Darmstadt, Hesse, Germany). Safflower seed oil, Tween 80, Span 85, and *α*-tocopherol were purchased from Sigma-Aldrich (Steinheim, North Rhine-Westphalia, Germany). Lecithin Lipoid S75 was purchased from Lipoid GmbH (Ludwigshafen, Rhineland-Palatinate, Germany). Tween 85 was purchased from Fluka Chemie GmbH (Buchs, St. Gallen, Switzerland), and glycerol was purchased from J.T. Baker (Phillipsburg, NJ, USA). Acetonitrile (HPLC grade) and simulated lung fluid were purchased from Fisher Scientific (Loughborough, UK) and R&M Chemicals (Petaling Jaya, Selangir, Malaysia), respectively. Palm kernel oil ester (PKOE) was synthesized directly in our lab using the enzymatic alcoholysis method [46]. Deionized water was obtained using the Milli-Q gradient water system (Billerica, MA, USA). All chemicals were used as received (AR grade) without additional purification unless otherwise stated.

### 3.2. Nanoemulsion Preparation

In general, an oil phase containing lecithin was stirred in a mixture of PKOE and safflower seed oil (1:1, *w*/*w*) at 250 rpm using a magnetic hotplate stirrer (C-MAG HS 7, IKA^®^, Wilmington, NC, USA) for 30 min at 40 °C. 1 mM of the active ingredient and Tween 85 and Span 85 (9:1, *w*/*w*) was added into the mixture and stirred for another 30 min or until all the ingredients were fully dissolved. Approximately 0.05 wt% of α-tocopherol was then added into the mixture and stirred for 5 min at 40 ± 1 °C. The prepared oil phase was then slowly added dropwise to an aqueous phase containing deionized water and glycerol and stirred at 350 rpm with an overhead stirrer (Stirrer RW16 Basic IKA^®^, Wilmington, NC, USA) for 30 min to form a pre-mixed emulsion. The emulsion was homogenized further using a high shear homogenizer (PT3100, Kinematica AG, Lucerne, Switzerland) at 15 min at 12,000 rpm.

### 3.3. Experimental Design

The effect of five independent variables, including oils (wt%) (A), lecithin (wt%) (B), Tween 85 and Span 85 (9:1) (wt%) (C), glycerol (wt%) (D), and water (wt%) (E), on the response variables of particle size and volume median diameter (VMD) was studied. The design matrix was generated based on the D-optimal criterion to select the candidate points using the Design-Expert software (v. 7, Stat-Ease Inc., Minneapolis, MN, USA) with a total of 24-candidate experiments. The upper (*Uj*) and lower (*Lj*) limits for each formulation components (*Xj*) were restricted to a specific range, to prevent the process from covering the entire simplex area [47]. In this design, the general form of constraints is as follows: *Lj* ≤ *Xj* ≤ *Uj*, where Σ *Xj* = 1. The lower and upper limits for each formulation independent variables were determined from a previous study [15], as summarized in Table 6.

### 3.4. Evaluation of the Fitted Models

To model the influence of formulation components (independent variables) towards the responses (dependent variables), three-dimensional (3D) surface response and contour plots were constructed by fitting the experimental data to the polynomial equations via multiple regression analysis. The analysis of variance (ANOVA) was conducted to evaluate the significance of coefficients between the variables and to generate the polynomial equation based on the fitted model. Three random formulations and a recommended optimum formulation for each DTX- and CCM-loaded nanoemulsion were subjected for validation of the generated model by comparing the predicted response value against the obtained value. An optimized formulation was suggested based on the smallest value of the response variable at a minimal amount of independent variables. The percentage of residual standard error (RSE%) for each response was determined using the following equation [47]:

(5) $$\mathrm{RSE}\left(\%\right)=\frac{\mathrm{Experimental}\;\mathrm{value}\;-\;\mathrm{Predicted}\;\mathrm{value}}{\mathrm{Predicted}\;\mathrm{value}}\;\times 100$$

### 3.5. Particle Size Distribution and ζ-Potential Determination

The mean particle size, polydispersity index (PDI), and *ζ*-potential of nanoemulsion formulations were determined using a Zetasizer (Nano ZS90, Malvern Instruments, Worcestershire, UK). Deionized water was used to dilute the sample (1:1000, *v*/*v*) to overcome the effect of multiple scattering [48]. The diluted sample was gradually loaded to full (~1.5 mL) in a folded capillary cell to prevent the formation of air bubbles before being inserted into the sample chamber. Measurements were carried out at controlled room temperature (25 ± 1 °C) after being equilibrated for 120 s to stabilize the cell area temperature. Each sample was measured three times, and the data were expressed in the mean.

### 3.6. Laser Diffraction Analysis

Laser diffraction method using a Spraytech (Malvern Instruments Ltd., Worcestershire, UK) was used to study the quality of the nebulized nanoemulsion aerosols. This method was reported to be consistent with many in-vivo findings on lung deposition [28,48]. An aliquot of 3 mL of nanoemulsion was added into a medication chamber of an OMRON MicroAIR nebulizer (NE-U22V1, Shimogyo-ku, Kyoto, Japan). The nebulizer was positioned perpendicular to the Spraytech laser lens axis and 20 mm away from the laser beam. The sample was then nebulized at 15 L/min flow rate by adjusting the suction of the vacuum pump located on the opposite side of the instrument. This suction is essential to make the aerosol pass through the laser beam at a normal inspiratory flow rate for aerosol size analysis.

The data were then analyzed using RTSizer software (v. 5.51, Malvern Instruments Ltd. Worcestershire, UK). This was done to determine the nanoemulsion aerosolization properties, including the DV_50_ (50% of the undersized particle distribution curve) and also known as the volume median diameter (VMD), DV_10_ (10% undersize particle distribution curve), DV_90_ (90% undersize particle distribution curve), and span which indicated how far the 10% and the 90% points were apart, normalized with the midpoint. The measurements were conducted in triplicate, and the mean data were presented.

### 3.7. pH Measurement

The pH measurement of the optimized nanoemulsions was measured using a pH meter (Delta 320, Mettler Toledo, Ota-ku, Tokyo, Japan). An electrode with a glass membrane that is sensitive to hydrogen ions was used to measure the pH value. The value was dependent on the hydrogen ion concentration, which was used to determine the acidity of the formulation [49]. The calibration of the pH meter was carried out using pH 4, 7, and 10. The glass electrode was dipped into the sample without dilution, and the sample pH value was then recorded. The measurement was conducted in triplicate at ambient temperature (28 ± 1 °C).

### 3.8. Viscosity Measurement

The viscosity of the optimized nanoemulsions was determined directly via a viscometer (Brookfield DV-II+, Middleboro, MA, USA) without dilution. Spindle SC4-18 was attached to the viscometer, and 2 mL of nanoemulsion sample was added into the sample cup. The sample cup was then connected to the viscometer and adjusted until the hit point (the desired gap between cone and cup) was satisfactorily reached. The sample was incubated for 10 min at 25 ± 1 °C to reach equilibrium, and the viscosity was measured in centipoise (cP). Upon rotation of the spindle, the viscous sample moved counter to the spindle wall. The viscosity was quantified based on the deflection amount of a calibrated spring. The measurement was repeated in triplicate, and the mean of viscosity value was calculated.

### 3.9. Osmolality Measurement

The osmolality value of the optimized nanoemulsions was determined using an osmometer (model 3320, Advance Instruments, Norwood, MA, USA) according to the freezing point principle. The osmometer was calibrated with a reference standard 100 and 3000 mOsm/kg (Advance Instruments, USA) at room temperature (25 ± 1 °C). Approximately 0.25 mL of sample was aspirated into the sampler tip, and the sampler was placed into the instrument’s cradle and then inserted into the system. The measurement was analyzed in triplicate, and the average of the osmolality value was calculated.

### 3.10. Transmission Electron Microscope Analysis

The morphology of freshly prepared optimized nanoemulsions was analyzed using TEM (H7100, Chiyoda-ku, Tokyo, Japan). The conventional negative staining method using uranyl acetate (1%, *w*/*v*) was applied. The samples were diluted 50 times with deionized water and vortexed for 10 s to prevent particle agglomeration. A 300 mesh formvar coated grid, stabilized with evaporated carbon film (FCF300-Cu, Radnor, PA, USA), was placed on a sample droplet and incubated at room temperature (28 ± 1 °C) for 5 min. Uranyl acetate solution was used to negatively stain the copper grid by placing the grid on the solution, and it was incubated for another 5 min at room temperature. Then, filter paper was used to remove excess uranyl acetate solution. The grid was then air-dried in a petri dish for 24 h before being analyzed.

### 3.11. In Vitro Deposition Test

The inhalation performance of both the DTX and CCM-loaded nanoemulsions (DTX_opt_ and CNE_opt_) was tested using an eight-stage Anderson cascade impactor (ACI) (Copley Scientific Ltd., Nottingham, UK) based on a method described by Arbain et al. [11]. Briefly, the ACI fitted with an adapter, mouthpiece, inlet cone, induction port, and stage 0 to filter was attached to a vacuum pump and operated at a 15 L/min flow rate to simulate the normal respiration of a healthy adult [28]. An aliquot of 3 mL DNEopt was added into the medication chamber of the OMRON MicroAIR nebulizer and was nebulized continuously into the ACI until dry. The deposited drug on each stage of the ACI together with the medication chamber, adapter, mouthpiece, inlet cone and induction port were collected. The steps were repeated with CNE_opt_, and the test was performed in triplicate.

The drug content in the collected samples was then analyzed by high-performance liquid chromatography (HPLC) (Waters 1525, Milford, MA, USA) using a C_18_ column (150.0 × 4.6 mm) (Eclipse plus, Agilent Technologies, Santa Clara, CA, USA), which was filled with particles size of 5 μm. The mobile phase ratio for detection of the DTX and CCM was 50:50 (*v*/*v*) and 36:64 (*v*/*v*), respectively, comprising filtered acetonitrile and deionized water at pH 3. The analysis was performed at a flow rate of 1 mL/min and an injection volume of 10 μL, at a detection wavelength of 228 nm for DTX, and 430 nm for CCM, using a UV/Vis detector. The polyvinylidene difluoride (PVDF) with a 0.45 µm pore size was used to filter the collected samples prior to injection.

The percent dispersed (PD) was calculated in percentage by dividing the total amount of drug collected from all parts of the ACI, including the inlet cone, induction port, adapter, mouthpiece, and stage 0 to filter, also known as the emitted dose (ED), over the total dose (TD) loaded in the nebulizer. The percent inhaled (PI) was calculated by dividing the total drug content in stages 0 to 7, over the TD. The mass median aerodynamic diameter (MMAD) was determined at the 50th percentile on the cumulative particle size distribution curve. In contrast, the geometric standard deviation (GSD) was determined by dividing the square root of the size obtained at the 84.13th percentile, over the square root of the size obtained at the 15.87th percentile. The fine particle fraction (FPF) was calculated in percentage by dividing the total amount of drug deposited on stage 4 to filter, over the ED.

### 3.12. In Vitro Drug Release Study

A dialysis membrane diffusion method [50] was utilized to study the in vitro drug release from the optimized nanoemulsion systems. Simulated lung fluids (SLF) at pH 6.5 and 7.4 comprising 0.2% (*w*/*v*) Tween 80 were used as dissolution media to simulate the lung interstitial fluid in tumor (pH 6.5) and healthy (pH 7.4) environments. Cellulose dialysis tubing with 12 kDa molecular weight cut-off (Fisherbrand^TM^, Loughborough, UK) was loaded with DNE_opt_ equivalent to 2 mg of DTX. The dialysis tube was immersed in the dissolution media at 37 ± 1 °C and continuously stirred using a magnetic bar at 250 rpm. An aliquot of 1 mL sample was collected at specified time intervals and was replaced by an equivalent volume of fresh medium to maintain the sink conditions. The amount of DTX in the collected samples was then determined using the HPLC method, as outlined in Section 3.11. The methods were repeated with CNE_opt,_ and the analysis was conducted in triplicate. The average of the drug release content at each time interval was then calculated.

## 4. Conclusions

The present study highlights the D-optimal mixture experimental design (MED) as being a potent tool to study optimization, which involved in the formulation of DTX and CCM-loaded nanoemulsions to attain small particle size and VMD. In general, the MED model predicted that the mean particle size of the nanoemulsion systems was directly proportional to the oil content and was inversely proportional to the amount of lecithin, Tween 85:Span 85 (9:1, *w*/*w*) and glycerol, while the VMD of the nanoemulsion systems was directly proportional to the oil and lecithin, and was inversely proportional to the Tween 85:Span 85 (9:1, *w*/*w*), and glycerol concentrations. The observed value of the suggested optimum formulations for particle size was in good agreement with the predicted value (RSE < 1.6%), which displayed better performance in model fitting than the VMD (RSE < 3.8). The optimized formulations exhibited desirable pH, viscosity, and osmolality attributes for pulmonary administration. The nebulized formulations exhibited a high percentage of fine particle fractions (FPF > 75%) and were predicted to mainly deposit in the lower airways of the lungs with an aerodynamic diameter less than 5.5 μm. These characteristics are responsible for making the optimized nanoemulsion system a great potential candidate for the delivery DTX and CCM via the pulmonary route.

## Figures and Tables

**Table 1:** Candidate points of D-optimal mixture experimental design together with the obtained particle size and aerosol size (VMD) of the docetaxel (DTX)- and curcumin (CCM)-loaded nanoemulsions.

Run	Components (wt%)					Particle Size (nm)		VMD (µm)	
	A	B	C	D	E	DTX	CCM	DTX	CCM
1	4.00	2.00	1.00	1.50	91.50	114.41	118.38	5.19	5.28
2	4.00	3.00	1.00	3.00	89.00	113.12	113.80	5.33	5.22
3	4.00	3.00	2.00	3.00	88.00	101.88	98.49	5.13	5.26
4	5.00	2.50	1.50	2.25	88.75	102.93	106.76	5.43	5.31
5	6.00	2.00	1.00	3.00	88.00	103.16	119.92	5.24	5.21
6	6.00	2.00	1.50	1.50	89.00	106.14	103.90	5.31	5.45
7	6.00	2.50	2.00	1.50	88.00	96.92	94.55	5.34	5.15
8	4.00	2.00	2.00	1.50	90.50	103.11	109.21	5.15	5.11
9	6.00	3.00	1.00	1.50	88.50	104.55	120.01	5.47	5.15
10	6.00	3.00	1.50	3.00	86.50	105.32	105.42	5.41	5.32
11	5.00	3.00	2.00	1.50	88.50	94.59	97.26	5.32	5.50
12	6.00	3.00	2.00	2.25	86.75	96.48	97.19	5.27	5.10
13	6.00	2.00	2.00	3.00	87.00	99.31	102.72	5.22	5.17
14	4.00	2.00	1.50	3.00	89.50	108.50	113.11	5.11	5.37
15	4.00	3.00	1.50	1.50	90.00	109.07	113.07	5.29	5.24
16	5.00	2.00	1.00	1.50	90.50	108.71	112.76	5.27	5.20
17	5.00	3.00	1.00	3.00	88.00	107.16	113.31	5.55	5.10
18	5.00	2.00	2.00	3.00	88.00	100.11	102.43	5.12	5.31
19	5.00	3.00	1.00	1.50	89.50	106.08	118.04	5.34	5.25
20	5.00	2.00	2.00	1.50	89.50	100.77	98.72	5.14	5.23
21	5.00	2.50	1.50	2.25	88.75	102.89	107.57	5.22	5.28
22	5.00	2.50	1.50	2.25	88.75	103.88	106.32	5.27	5.11
23	5.00	2.50	1.50	2.25	88.75	103.76	106.28	5.29	5.36
24	4.00	3.00	2.00	3.00	88.00	102.52	97.51	5.30	5.39

A: amount of PKOE: Safflower seed oils (1:1); B: amount of lecithin; C: amount of Tween 85:Span 85 (9:1); D: amount of glycerol; E: amount of water.

**Table 2:** Analysis of variance of the fitted quadratic model for particle size and the linear model for aerosol size (VMD) of the DTX- and CCM-loaded nanoemulsion.

Particle Size	DTX			CCM		
	Mean Square	*F*-Value	*p*-Value	Mean Square	*F*-Value	*p*-Value
Model	37.87	81.30	<0.0001	100.58	401.53	<0.0001
AB	3.05	6.54	0.0308	2.82	11.25	0.0085
AC	21.06	45.21	<0.0001	18.42	73.55	<0.0001
AD	4.32	9.27	0.0139	3.76	15.03	0.0038
AE	13.07	28.06	0.0005	43.32	172.92	<0.0001
BC	0.95	2.03	0.1879	13.67	54.57	<0.0001
BD	0.80	1.72	0.2220	3.63	14.48	0.0042
BE	1.43	3.07	0.1136	1.49	5.95	0.0374
CD	23.14	49.67	<0.0001	0.17	0.68	0.4320
CE	26.08	55.99	<0.0001	2.40	9.60	0.0128
DE	1.00	2.15	0.1762	6.24	24.92	0.0007
Residual	0.47	-	-	0.25	-	-
Lack of fit	0.60	2.28	0.2219	0.12	0.28	0.9043
Pure error	0.27	-	-	0.42	-	-
**VMD**						
Model	0.05	10.11	0.0001	0.05	15.13	<0.0001
Lack of fit	<0.01	0.34	0.9431	<0.01	0.56	0.8135
Pure error	<0.01	-	-	<0.01	-	-

**Table 3:** Actual and predicted response values for randomized and optimized formulations.

Formulation	Components (wt%)					Particle Size (nm)		RSE (%)	VMD (μm)		RSE (%)
	A	B	C	D	E	Act.	Pre.		Act.	Pre.	
**DTX-Loaded Nanoemulsion**											
DNE_1_	5.50	2.60	1.60	2.20	88.10	103.5	101.5	1.97	5.45	5.31	2.64
DNE_2_	4.50	2.40	1.40	2.30	89.40	107.3	106.1	1.13	5.39	5.22	3.26
DNE_3_	5.20	2.25	1.60	2.20	88.75	102.4	102.8	0.39	5.41	5.26	2.85
DNE_opt_	6.00	2.50	2.00	2.50	87.00	95.8	97.2	1.44	5.48	5.28	3.79
**CCM-Loaded Nanoemulsion**											
CNE_1_	5.20	2.70	1.70	2.00	88.40	103.1	102.6	0.49	5.44	5.31	2.45
CNE_2_	4.80	2.70	1.30	2.10	89.10	113.2	110.8	2.17	5.34	5.26	1.52
CNE_3_	4.50	2.30	1.60	2.40	89.20	108.8	110.5	1.54	5.25	5.17	1.55
CNE_Opt_	6.00	2.50	2.00	2.50	87.00	96.9	98.4	1.52	5.40	5.32	1.50

RSE: residual standard error; Act: actual; Pre: predicted.

**Table 4:** Physicochemical characteristics of the optimized formulations.

Characteristics	DNE_Opt_	CNE_Opt_
Particle size (nm)	95.80 ± 1.13	96.94 ± 0.88
Polydispersity index	0.25 ± 0.02	0.22 ± 0.02
*ζ*-potential (mV)	−36.23 ± 1.25	−33.40 ± 2.11
pH	7.12 ± 0.01	7.14 ± 0.02
Viscosity (cP)	2.22 ± 0.09	2.05 ± 0.10
Osmolality (mOsm/kg)	297.00 ± 1.00	283.00 ± 1.00

DNE_Opt_—optimized DTX-loaded nanoemulsion; CNE_Opt_—optimized CCM-loaded nanoemulsion.

**Table 5:** Aerosolization and inhalation performances of optimized formulations.

Characteristics	DNE_Opt_	CNE_Opt_
**Spraytech Laser Diffraction Method**		
DV_10_ (μm)	2.50 ± 0.05	2.38 ± 0.08
DV_50_ (μm)	5.48 ± 0.14	5.40 ± 0.16
DV_90_ (μm)	11.48 ± 0.83	11.53 ± 0.92
Span	1.64 ± 0.12	1.69 ± 0.14
**Anderson Cascade Impaction Method**		
PD (%)	70.82 ± 10.66	73.89 ± 4.03
PI (%)	70.13 ± 10.61	72.67 ± 3.89
MMAD (μm)	3.19 ± 0.15	3.08 ± 0.15
GSD	1.71 ± 0.08	1.67 ± 0.15
FPF (%)	76.85 ± 6.01	83.30 ± 2.81

PD: percent dispersed; PI: percent inhaled; MMAD: median mass aerodynamic diameter; GSD: geometric standard deviation; FPF: fine particle fraction.

**Table 6:** Limits of the formulation of independent variables.

Independent Variable, *Xj*	Lower Limit, *Lj*	Upper Limit, *Uj*
PKOE and Safflower oils (1:1), A	4.0	6.0
Lecithin, B	2.0	3.0
Tween 85 and Span 85 (9:1), C	1.0	2.0
Glycerol, D	1.5	3.0
Water, E	86.0	91.5

A: PKOE: Safflower seed oils (1:1); B: lecithin; C: Tween 85:Span 85 (9:1); D: glycerol; E: water.

## Abbreviations

ACI	Anderson cascade impactor
CCM	Curcumin
DTX	Docetaxel
ED	Emitted dose
FPF	Fine particle fraction
GSD	Geometric standard deviation
MED	Mixture experimental design
MMAD	Mass median aerodynamic diameter
PD	Percent dispersed
PDI	Polydispersity Index
PI	Percent inhaled
PKOE	Palm kernel oil ester
RSE	Residual standard error
SLF	Simulated lung fluid
TD	Total dose
TEM	Transmission electron microscopy
VMD	Volume median diameter
